# Comparative case studies in integrated care implementation from across the globe: a quest for action

**DOI:** 10.1186/s12913-019-4661-5

**Published:** 2019-11-27

**Authors:** Nicole A. Stadnick, Euan Sadler, Jane Sandall, Cristina Fernandez Turienzo, Ian M. Bennett, Jeffrey Borkan, Bibilola Oladeji, Oye Gureje, Gregory A. Aarons, Marisa Sklar

**Affiliations:** 1 Department of Psychiatry, University of California San Diego, La Jolla, USA; 2 Child and Adolescent Services Research Center, San Diego, USA; 3 Health Service & Population Research Department, Centre for Implementation Science, King’s College London, London, UK; 4 Department of Nursing, Midwifery and Health, School of Health Sciences, Faculty of Environmental and Life Sciences, University of Southampton, Southampton, UK; 5 Department of Women and Children’s Health, School of Life Course Sciences, Faculty of Life Sciences & Medicine, King’s College London, London, UK; 6 Department of Family Medicine, University of Washington, Seattle, USA; 7 Department of Psychiatry and Behavioral Sciences, University of Washington, Seattle, USA; 8 Department of Global Health, University of Washington, Seattle, USA; 9 Department of Family Medicine, Brown University, Providence, USA; 10 Department of Psychiatry, College of Medicine, University of Ibadan, Ibadan, Nigeria

**Keywords:** Integrated care, Global healthcare, Multiple case study, EPIS framework

## Abstract

**Background:**

Integrated care is the coordination of general and behavioral health and is a highly promising and practical approach to improving healthcare delivery and patient outcomes. While there is growing interest and investment in integrated care implementation internationally, there are no formal guidelines for integrated care implementation applicable to diverse healthcare systems. Furthermore, there is a complex interplay of factors at multiple levels of influence that are necessary for successful implementation of integrated care in health systems.

**Methods:**

Guided by the Exploration, Preparation, Implementation, Sustainment (EPIS) framework (Aarons et al., 2011), a multiple case study design was used to address two research objectives: 1) To highlight current integrated care implementation efforts through seven international case studies that target a range of healthcare systems, patient populations and implementation strategies and outcomes, and 2) To synthesize the shared and unique challenges and successes across studies using the EPIS framework.

**Results:**

The seven reported case studies represent integrated care implementation efforts from five countries and continents (United States, United Kingdom, Vietnam, Israel, and Nigeria), target a range of clinical populations and care settings, and span all phases of the EPIS framework. Qualitative synthesis of these case studies illuminated common outer context, inner context, bridging and innovation factors that were key drivers of implementation.

**Conclusions:**

We propose an agenda that outlines priority goals and related strategies to advance integrated care implementation research. These goals relate to: 1) the role of funding at multiple levels of implementation, 2) meaningful collaboration with stakeholders across phases of implementation and 3) clear communication to stakeholders about integrated care implementation.

**Trial registration:**

Not applicable.

## Introduction

In recent decades, integrated care has received increased attention globally. Policymakers, providers, payers, and healthcare consumers propose that integrated care holds promise in facilitating healthcare improvements [1, 2]. A global aging population and advances in medical science and technology mean that individuals are living longer, but often with increased incidence and prevalence of long-term conditions [3] and multimorbidity. Despite these changes in healthcare needs, many healthcare systems focus on acute care needs [4–7]. In addition, policymakers, providers, consumers, and payers agree that healthcare systems largely reward quantity of services delivered at the expense of higher quality care [8]. While significant reform is often required for successful integration, there is no national or international consensus regarding the best guidelines or set of implementation strategies for integrated care efforts [9]. To address this lack of consensus, we used a multiple case study design and the Exploration, Preparation, Implementation, Sustainment (EPIS) framework [10], a widely cited implementation science framework [11], to prioritize goals and strategies to advance integrated care implementation research generalizable across countries and healthcare systems.

Over 175 definitions for integrated care exist [12], as well as different models of integrated care [13]. For this study, we conceptualize integrated care similar to Mur-Veeman and colleagues (2003) as an organisational process of coordination across services or systems (e.g., primary and mental healthcare; health and social care) seeking to achieve seamless and continuous care, tailored to patients’ needs, and based on a holistic view of the patient [14] (i.e., attending to the whole-person, including behavioral health needs, chronic health conditions or comorbidities). Integrated care models have shown to produce beneficial impacts internationally [15] but the implementation strategies, implementation processes and implementation outcomes of integrated care have not been thoroughly investigated.

A complex interplay of factors integral to integrated care implementation exist. A commentary paper discussing the implementation of integrated care noted several factors impacting implementation [16]. Barriers of integrated care implementation include “operational complexity, regulatory challenges, unclear financial attribution, and cultural inertia [16].” For successful integrated care implementation, the authors argue that long-term plans with adequately protected support and funding must be present. Additionally, integrated care models in healthcare systems such as the UK have had limited success due to a lack of sustained project management support—restricting implementation efforts to short-term projects [17].

This complexity necessitates considering multiple levels of influence including patients who access healthcare, providers who deliver care, organizations that provide the infrastructure for healthcare, and policymakers influencing the funding and processes of care delivery [11]. The EPIS framework [10] was selected to guide our case study data extraction, results and discussion of integrated care implementation in different countries. This framework was chosen over other frameworks because it is multilevel and addresses phases and processes to maximize the uptake, implementation, and sustainment of integrated care programmes. The EPIS framework delineates outer context (i.e., system-level), inner context (i.e., organizational, provider, patient), bridging (i.e., interface between outer and inner contexts) and innovation (e.g., characteristics of integrated care) factors from adoption to sustainment [18]. A primary objective of EPIS is to maximize the “fit” between the innovation and the implementation service context(s). “Fit” can be facilitated by including active, well-defined community-academic partnerships that include a range of relevant stakeholders (e.g., patients, providers, organizational leaders) [19].

The EPIS framework was used to pursue two objectives. The first was to highlight current integrated care implementation efforts through international case studies targeting different healthcare systems, patient populations and implementation outcomes. The second objective was to synthesize the shared challenges and successes across our case studies and propose an agenda of priorities and critical implementation strategies for integrated care implementation generalizable across countries and healthcare systems.

## Method

### Design

This study used an explanatory multiple case study design to illuminate shared and unique implementation processes present in contemporary integrated care implementation efforts across different countries and contexts. The core research team (NS, ES, MS) invited researchers targeting integrated healthcare implementation in North America (United States of America [USA]), Europe (United Kingdom [UK]), South America (Peru), Asia (Israel) and Africa (Nigeria) to contribute a case study. The intention was to include case studies that represented unique implementation efforts across the EPIS phases, that focused on heterogenous patient populations, and targeted a range of outer and inner context factors to highlight the unique, and at times, consistent challenges and successes at different points of integrated care implementation. This approach incorporates the four main features of multiple case study designs [20]: 1) a conceptual framework (EPIS) to provide a superordinate structure, 2) a sampling plan (described below) to highlight a breadth of integrated care implementation examples, 3) procedures for collecting data about each individual case study and 4) a cross-case study analysis using qualitative synthesis procedures. Each implementation effort (*N* = 7) is conceptualized as a “case” [21, 22] in line with case study methodology recommendations described by Small [23].

### Procedures

After identifying lead research investigators who agreed to contribute a case study, the core research team and investigators (NS, ES, JS, CFT, IB, JB, MS) completed a 1–2 page summary of the implementation effort based on a shared template used to collect, organize, and communicate characteristics for each case study. See Table 1 for the shared case study template. Case studies were analyzed by the core research team in an iterative manner using a template organizing style [24]. The full text of each submitted case study was read by each of the core research team members to gain familiarity with the content, and broad themes were isolated from theoretical and conceptual considerations. The EPIS framework [10] was used to guide interpretation of each study’s design and results. The core research team held a total of three, 60–75 min, meetings to discuss “chunks” [24] of text, further the analysis, and finalize interpretation.

**Table 1:** Shared Case Study Template

Topic	Sub-Sections
Background on integrated care approach (es) applied in implementation effort	1. Intended care setting(s) of integration.
	2. Intended patient population(s).
	3. Intended goals or effects of the integrated care approach.
Implementation methods used in implementation effort	4. EPIS phase(s) targeted.
	5. Key outer and inner context factors targeted.
	6. Implementation strategies used or proposed.
	7. Implementation outcomes targeted.
Challenges, successes, and/or lessons learned from implementation effort	8. If available, supporting data to illustrate challenges, successes or lessons learned.

### Sampling

Because a random sampling approach is not recommended for multiple case study designs [21, 23], we elected to use a snowball sampling strategy to identify unique and cross-cutting implementation themes across heterogeneous case examples [25]. The core research team convened over several virtual meetings to explore a network of integrated care implementation experts for potential contribution to this study. The network was reviewed paying particular attention to heterogeneity of integrated care implementation endeavors in an effort to uncover and synthesize emerging implementation themes across varying contexts. Thus, seven case studies were identified in a progressive fashion that represented integrated care implementation efforts across different continents and countries, and were at different stages of the EPIS framework. As each international integrated care implementation effort was identified, the core research team invited representatives from the effort to contribute to this study—all of whom agreed to contribute. Our resulting case studies comprise diverse integrated care implementations targeting varied populations and health concerns, and varying healthcare delivery and financing systems. The seven case studies included are summarized in Table 2 and described in the following section. For the full text of each case study submitted along with illustrative quotations, please see Additional file 1.

**Table 2:** Characteristics of case studies

Case Study: Target Population and Health Outcome	Country	Healthcare Financing Structure		EPIS Phase(s) Targeted			
		Single Payer	Multiple Payer	E	P	I	S
1. Integrated Care for Older Adults with Frailty	United Kingdom	X		X	X		
2. Access to Integrated Care Tailored for Children with Autism Spectrum Disorder	United States		X	X	X	X	
3. Perinatal Depression Screening and Treatment	Vietnam	X		X	X	X	
4. Integrated Care Following Mental Health Insurance Reform	Israel	X				X	
5. Scaling up Care for Perinatal Depression in Primary Care	Nigeria		X			X	
6. Midwifery Continuity of Care Model to Reduce Preterm Birth	United Kingdom	X				X	X
7. Patient-Centered Medical Home Model for Adults and Children to Improve Health and Experience at a Reduced Cost	United States		X				X

*Note.* Please refer to the Additional File for greater details about each case study

## Results

Case studies are presented in order of their EPIS phase of implementation. Although implementation efforts may include activities across multiple phases, case studies representing efforts that are in the early stages of the EPIS framework are presented first, while case studies that are further along in implementation are presented last. Outer and inner context factors, as well as bridging and innovation factors, for integrated care implementation are discussed.

### Integrated Care for Older Adults with frailty in South London, UK (Exploration-Preparation)

To meet the healthcare needs of growing numbers of older people, this project targets implementation of a co-designed integrated care model for community dwelling older adults with frailty [26] in South London, UK. Currently evidence of the impact of integrated care for older adults with frailty is equivocal [27]. Different models of integrated care exist, but it is not known what models or formulations of components of integrated care are most effective for this patient population [27]. EPIS inner and outer context factors have influenced the design and early adoption of integrated care for older adults with frailty. Key outer context factors driving implementation included the role of national and local policy and funding to improve integrated care delivery for cohorts of patients at high risk of hospital admission [28], as well as early stakeholder engagement and relationship building with local care providers, service users and caregivers. Key inner context factors included organizational capacity characteristics (i.e. role specialization, knowledge skills, expertise, values) and leadership qualities required to enable early adoption and identification of leaders in the system to champion adoption and delivery of integrated care.

A number of challenges, successes and lessons learned with regard to implementation of integrated care for older adults with frailty were identified through a multi-stakeholder qualitative study. First, providers working in different parts of the healthcare system shared an understanding that integrated care for older adults with frailty involves different providers working in effective multi-disciplinary teams across different care organizations and sectors to deliver patient-centered, holistic, and coordinated health and social care. A number of care professionals perceived that there were improved relationships between providers working in health and social care. Despite these improved relationships, there persisted limited care coordination and teamwork of providers across health, social and voluntary care sectors. Most service users and caregivers demonstrated difficulty conceptualizing integrated care. Some understood it as improved coordination of health and social care services, whilst others viewed integrated care as continuity of care with a trusted professional who knew them well and had the right information and resources to access and navigate the system. Perceived barriers to integrated care implementation among stakeholders related to organizational or system coordination factors (e.g. lack of pooled budgets, limited co-location of teams, limited access to shared patient records), and individual characteristics (e.g. patient complexity, variations in attitudes of managers, leaders and frontline staff).

### Access to integrated care tailored for children with autism Spectrum disorder in California, USA (Exploration-Implementation)

The goal of the “Access To Tailored Autism INtegrated Care” (ATTAIN) [29] study is to adapt and implement a behavioral health integrated care model between pediatric primary care and mental healthcare, for children with autism spectrum disorder (ASD). While integrated care is not standard practice in pediatrics, there is growing support for pediatric integrated health care approaches to facilitate addressing unmet specialty health care needs, including mental health [30, 31]. The ATTAIN study is currently in the Exploration phase and will span through Preparation to the Implementation phase. The key inner context factors are being measured through a mixed-methods needs assessment involving organizational healthcare leaders, pediatric primary care providers, and caregivers of children with ASD. Informed by these inner context factors, the primary implementation strategy applied to ATTAIN implementation is establishing a community-academic partnership to promote successful implementation and pediatric primary care provider training in tailored mental health screening and referral practices established by the ATTAIN model of integrated care. Based on preliminary qualitative data, several successes, challenges, and lessons learned have been illuminated. A key driver of ATTAIN implementation is the degree to which integrated care already exists and the ability to adapt components of healthcare delivery within the limitations of the organizational structure of the healthcare organization. This is consistent with the EPIS notion that adaptation may be needed at the outer or inner context, or to the integrated care model (the innovation) itself. However, the current healthcare landscape in primary care is not setup to support or incentivize pediatric primary care providers to attend to mental health concerns. Together, these needs assessment results highlight the dynamic interplay between outer and inner context factors that need to be proactively considered at the early stages of implementation.

### Perinatal depression screening and treatment in Can Tho, Vietnam (Exploration-Implementation)

This project aims to implement an integrated care approach (collaborative care) to improve screening and care for common perinatal mental health disorders in Vietnam, a lower-middle income country with a significant focus by national policy makers on the delivery of healthcare through public health systems. Perinatal mental health disorders are more prevalent in Vietnam than in high-income countries, and integrating mental health care with primary perinatal health care activities is essential [32]. This project is currently in the Exploration and Preparation phases of the EPIS framework. The overarching implementation strategy used for this project was a community-academic partnership through a participatory developmental approach. The key outer context factor that was an implementation driver was the National Mental Health Initiative for primary care, which identified depression as a target. The key inner context factors targeted were organizational characteristics of the clinics (e.g., infrastructure to support collaborative care, implementation climate, volume of prenatal and pediatric patients) and individual characteristics (e.g., training and professional background of providers, knowledge of perinatal depression). Results from descriptive and qualitative data indicated that there was a lack of knowledge of the symptoms of perinatal depression but, once reviewed, providers indicated recognition of this condition as common. There was a high level of perceived need and alignment with the goals of the health settings but a lack of training and procedures to allow for screening and care of perinatal depression.

Several implementation themes have characterized this integrated care effort. A critical implementation driver was the existing federally-supported effort to initiate screening and treatment for depression. This national policy initiative was an opportunity to leverage regional resources for the pilot implementation study. The primary challenge to implementation was identification of additional funding sources for the participation of external experts and health services researchers to support the pilot and evaluation.

### Integrated care following mental health insurance reform in Israel (Implementation)

This project focuses on implementation of integrated behavioral healthcare in Israel following a mental health insurance reform in 2015 that transferred responsibility for the provision of mental healthcare from the Israeli Ministry of Health to the four major health maintenance organizations (HMO) health plans [33]. While the quality of primary care provided by the Health Plans has been found to be better than similar plans in the United States [34], preliminary evaluations of the impact of mental health insurance reform indicate continued inefficiencies integrating mental health care into primary care [35]. We highlight one clinic’s implementation effort, the Tivon General Sick Fund (*Clalit*) clinic, in northern Israel. Numerous outer and inner context factors influenced implementation of integrated behavioral health in the Tivon clinic. The primary outer context influence was the financial restructuring of mental health services through the 2015 mandate. Stakeholder commitment and engagement, together with organizational inertia, were key inner context factors aiding implementation. The HMOs hired trained personnel (e.g., medical staff, psychiatrists, therapists, social workers), many of whom had previously worked for the Ministry of Health. In addition, the Tivon clinic designated a room for behavioral health providers to facilitate integration.

Throughout implementation in the Tivon clinic, several challenges and some successes emerged. While there was hope for increased communication and cooperation between the primary care providers and the behavioral health staff, this collaboration was never realized. There are two potential reasons for this unrealized hope: (1) the Tivon clinic was only provided with one psychiatrist despite promises of also being provided with a social worker and therapist, and (2) psychiatric services were only offered once every 2 weeks, precluding the psychiatrist from joining weekly staff meetings. Additional challenges remain regarding availability of services, largely associated with the concentration of mental health professionals in the large cities and inequities in distribution based on social economic and socio-political factors. Despite ongoing challenges, successes should be acknowledged. For example, there has been a transfer of responsibility for some of the less serious mental health illnesses from psychiatry to primary care. Additionally, relative integration of physical and mental health services has been achieved in some clinics across Israel [36–38], with the elimination of prior institutional barriers.

### Scaling up Care for Perinatal Depression in primary care, Oyo state, Nigeria (Implementation)

The Scaling up Care for Perinatal Depression for Improved Maternal and Child Health (SPECTRA) project used a task sharing approach to integrate care for perinatal depression into primary maternal care where the largest proportion of Nigerian women receive maternal and child health services. Integrating mental health services into routine primary/maternal care such that non-physician health care providers deliver the bulk of essential mental health care service under the support and supervision of physicians or psychiatric nurses (who are themselves supported by more highly trained mental health specialists at regional/state levels) is commonly agreed as the most effective way to bring care to those in need [39, 40]. The key outer context driver of implementation was heightened state policy attention to mental healthcare. This led to the development of a cascade training approach, where psychiatrists trained senior level primary healthcare workers who then provided training to frontline primary healthcare workers to implement the existing National Mental Health policy on maternal mental healthcare. Inner context factors impacting the implementation of integration include an initial resistance to change by frontline primary healthcare workers, limited knowledge of perinatal depression screening, high workload, and the absence of consistent leadership in the form of supportive supervision for the frontline providers to facilitate the delivery of evidence-based care for perinatal depression. Even though the cascade training resulted in measurable improvement in the knowledge and attitudes about depression, a persisting challenge was the low detection rates (14%) of perinatal depression following training. In response, a structured supportive supervision program and a depression screening tool were added at the clinics.

### Midwifery continuity of care model for women at increased risk of preterm birth in London, UK (Implementation-Sustainment)

Midwife-led continuity models of care have been demonstrated to provide greater benefits for women and babies, with no adverse effects, when compared to other models of care for women during pregnancy, birth, and early parenting [41]. This case study describes results from a hybrid type-2 effectiveness-implementation pilot trial [42] of a new midwife continuity of care model with rapid referral to a specialist obstetric preterm birth clinic for women at increased risk of preterm birth in South London, UK. During the Exploration and Preparation phases, several outer and inner context factors were identified to facilitate implementation of the integrated care pathway. Key outer context influences were the national maternal policy “Better Births” [43] to increase continuity of care, enhanced tariffs from local clinical commissioner groups, and the development of a robust network between midwifery services and other external organizations. In contrast, key outer context factors hindering implementation included mimetic pressure from competing organizations who had already implemented similar models of care. Key inner contextual factors that facilitated implementation included organizational commitment and a shared vision at local and national levels, as well as enhanced leadership and visibility. Inner context factors hindering implementation included a lack of tangible financial incentives, significant staffing shortages, and organizational disruption.

The identified inner and outer context factors informed selecting more than 20 evidence-based implementation strategies [44] (e.g., a local needs assessment, building a coalition to co-develop health programmes). The implementation of a midwifery continuity of care model involved a complex, large-scale transformation of the organisation of maternity care services. Key achievements have been the early and ongoing engagement with the commissioners of maternity services who have provided additional financial support for a clinical lead and commissioning the planned service in the contract with the hospital, the ongoing sustainability of the team and planned scale up after the research is completed. Others include contribution to national maternity policy to increase continuity models of care nationally and receipt of the NIHR South London Research Collaborative ‘*Most Innovative Collaboration*’ Award.

### Patient-centered medical home model for adults and children to promote health and wellbeing in Rhode Island, United States (Sustainment)

The provision of integrated care through implementation and sustainment of the Patient-Centered Medical Home Model (PCMH) of service delivery at the Family Care Center (FCC) at Memorial Hospital of Rhode Island, USA, provides an example of an integrated care implementation. The FCC offers integrated multidisciplinary services for patients across the lifespan. PCMHs have demonstrated effectiveness in cost savings by reducing hospital and emergency department visits, mitigating health disparities, and improving patient outcomes [45–50].

Several outer and inner context factors were key in facilitating the FCC’s progression into the Sustainment phase of PCMH implementation. Key outer context factors include a history of transdisciplinary, and legislative, initiatives to improve United States healthcare and patient health outcomes (e.g., Patient Protection and Affordable Care Act of 2010) [51]. Several inner context factors were also important in facilitating the FCC’s progression into the Sustainment phase. The Department of Family Medicine at Brown Medical School housed faculty members with leadership roles in national organizations in academic family medicine that facilitated the collaboration and knowledge sharing between the FCC and Patient-Centered Primary Care Collaborative regarding the future of family medicine. These faculty members then obtained grant funding to support the emerging PCMH movement in Rhode Island and enrolled the FCC in a statewide chronic care collaborative in 2003. Another important inner context factor in facilitating the FCC’s progression into the Sustainment phase of PCMH implementation is that Brown Medical School curriculum has been adapted to include additional didactic and experiential training on the PCMH.

While the FCC has been able to sustain the PCMH model of service delivery, areas for improvement reported by FCC faculty and resident physicians include ongoing challenges of limited time, feeling/being under resourced, limited staffing, and payment systems that do not adequately support the PCMH model. Despite these challenges, several successes deserve acknowledgement. Many faculty and resident physicians described the widespread knowledge of the PCMH within the FCC, and the integration of some structures and processes to facilitate integration, as successes.

## Discussion

A growing number of integrated care efforts have demonstrated both positive and equivocal outcomes [15]. To maximize effectiveness, integrated care approaches must be implemented with careful consideration of contextual factors that influence implementation. We used a multiple case study design to inform an agenda of priorities for ongoing and future integrated care implementation research. We first describe key outer, inner and bridging/innovation factors from the EPIS framework [10, 18] that emerged as common themes of integrated care implementation. We then propose an implementation agenda generalizable across populations, contexts, and settings that future research can address to improve integrated care.

### Outer context considerations

Five key outer context factors were identified across the case studies. (1) The first key shared outer context factor related to patient/client characteristics, specifically, the degree to which specified patient populations (e.g., patients at high risk for hospital admission) had been identified as the targets for integrated care and greater levels of socio-political support (e.g., through legislative attention or policy mandates) to improve care for these groups. (2) Secondly, leadership was critical for implementation, particularly the engagement of higher-level leaders and state authorities who can determine priorities for healthcare reform efforts. Recent work has shown that both strong system level and clinic level leadership predict EBP sustainment [52]. (3) The third important outer context factor was funding, which was seen to impact integrated care implementation in a number of ways. Certain healthcare payment models, such as the division between public and private healthcare funding in the USA, may not adequately support integrated care approaches. In these non-supportive funding structures, there is often inadequate time that can be reimbursed to address the holistic needs of the patient (either in person or behind-the-scenes collaboration/consultation). Supplemental and ongoing funding for implementation projects is challenging due to limited funds, higher contemporary competition for grants, and funding fluctuations and/or imbalances across systems. These constraints can have a particular impact on target populations with long-term conditions, who often present with complex health and social care needs. In addition, funding provisions for integrated care through national or local policy drives demonstrated system-level support for integrated care implementation. However, as illustrated in the case study in Israel following an insurance reform, a policy mandate can be a necessary but not sufficient implementation strategy to facilitate integrated care implementation. For example, in addition to legislative policy mandating change, there also needs to be a suitable, ideally nimble, funding infrastructure that both allows for integrated care delivery to be reimbursed and holds health systems accountable for funding integrated care.

(4) The fourth shared outer context factor related to the extent and nature of patient/client advocacy and involvement. Our case studies revealed that consensus from stakeholders about prioritizing integrated care implementation and the voice of service users are critical at all stages of implementation. National healthcare agencies like the US Agency for Health Research and Quality [53] and federal healthcare initiatives like the National HIV/AIDS Strategy [54] have developed divisions of priority populations with the intention of reducing disparities in care.

(5) Finally, inter-organizational environment and networks were identified as an important outer context factor shaping integrated care implementation. Many of the case studies mentioned the role of the electronic health record in facilitating (if shared across systems) or impeding (if incompatible) integration across service systems. Related to cross-service system communication, the extent to which primary and specialty care services were co-located, available and/or accessible to service users was an important consideration. In some cases, the extent of meaningful and collaborative communication was influenced by perceptions of the potential for loss of confidentiality or release of personal health information. This was particularly salient for mental health providers who may be reluctant to share patient records with other non-mental health providers due to the sensitive information about the client’s mental health experiences.

### Inner context considerations

Five inner context factors were particularly relevant across the case studies. (1) Individual characteristics of service providers related to their knowledge, education or professional training in integrated care, as well as their confidence as a provider of integrated care, were key inner context factors shared across cases studies. This was sometimes shaped by insufficient clarity about the roles and functions of providers involved in integrated care delivery. It was also shaped by service provider’s understanding and prioritization of healthcare needs of the population targeted. (2) Several organizational characteristics were important for adoption and implementation of integrated care approaches across different health populations. These included the integrated care implementation climate [55, 56] (defined as the extent to which there is a shared perception that integrated care is expected, supported and rewarded within an organization); communication between providers or staff within an organization to support integrated care; and variation in workforce readiness to implement models of integrated care. Many of these organizational characteristics have also been found to be influential in broader implementation efforts [57–59] of complex interventions in diverse practice settings and populations. A recent study has indicated that in organizations with a positive ‘molar climate’ (i.e. shared perceptions of how the workplace impacts personal wellbeing), stronger implementation climate longitudinally predicted use of evidence-based practices in behavioral health organizations [57]. That is, for effective implementation an organization should be functioning well and then strategic focus on climate for implementation will be more effective compared to organizations that are not functioning well. (3) Leadership is the third important inner context factor identified across case studies. A key consideration was the alignment across different levels of leadership within and across organizations in support of the goals of integrated care. (4) The extent to which quality improvement and/or fidelity monitoring (e.g., audit and feedback) was considered in implementation was the fourth key shared inner context factor. Two particular considerations related to this were the extent to which service user or patient-centered quality monitoring outcomes were used (e.g., satisfaction with integrated care implementation) and the role of patient registries or electronic health records to facilitate integrated care implementation or sustainment. A recent qualitative study has highlighted the requisite conditions of successful implementation of patient registries [60]. These conditions include an emphasis/interest in continuous quality improvement (QI mindset), sufficient resources to develop/maintain the registry, leadership support and key personnel who directly facilitated registry implementation, and whether a practice was part of a large health system.

(5) Finally, organizational staffing processes, like shifting employees’ roles and responsibilities to facilitate integrated care implementation and sustainment, emerged as a fifth shared inner context factor. This could take the form of transitioning nursing staff to nurse care managers to review high risk patient lists, outreach, and coordinate care. This factor also relates to individual characteristics of service providers and their attitudes towards shifting their roles and functions to facilitate integrated care.

### Bridging factors

There was one primary bridging factor identified as key to integrated care implementation across all the case studies. This bridging factor was establishing and involving a community-academic partnership [19] with the purposes of increasing knowledge, promoting buy-in and fostering engagement from a range of key informants/stakeholders (e.g., system/organizational leadership, health management consultants, providers, patients) involved in integrated care implementation. Our case studies, as well as the larger literature, underscore the significance of establishing and maintaining community-academic partnerships starting in the early phases of implementation (during the transition from usual care to integrated care) through the sustainment phase (when integrated care becomes the routine, standard of care) [61]. The process of establishing and maintaining community-academic partnerships affords the opportunity to combine the contributions of key community members/stakeholders who have practical expertise with the contributions of implementation researchers who have scientific experience to potentially increase the public health impact of integrated care implementation.

### Innovation factors

There was one primary innovation factor identified: the degree of fit between the integrated care innovation and the system(s), organisation, provider, and patient/client groups. Fit was shaped by several elements of the contextual implementation environment including: the magnitude of siloed care prior to integrated care implementation; the extent to which stakeholders perceived that integrated care was an aspiration but discordant with the reality of care delivery; the perception that the role and identity of physician is more narrowly focused on medical/acute conditions rather than the “whole” patient (a core principle of integrated care); the extent of (collaborative) communication between providers, leaders, stakeholders from different service systems; and service user characteristics, including the complexity of their healthcare needs for target populations (e.g., frail older adults, children with ASD).

### Global agenda to advance implementation of integrated care

Based on these outer, inner, bridging and innovation factors, we propose an agenda of three broad goals to advance research in integrated care implementation.

#### (1) Consider the role of funding at multiple levels of implementation

As with most implementation efforts in health service contexts, funding is a vital condition for successful transformation of healthcare delivery. At the outer context level, national and international research funding agencies can support the establishment of integrated care implementation by prioritizing funding for integrated care implementation research programmes, including funding for early and later phases of the implementation process. At the system-level, bridging strategies can reinforce priorities for integrated care implementation and can be communicated through incentives, mandates, and encouragement, and monitoring of national or local policy that communicates and reinforces the goal of adoption and implementation of integrated care approaches [2]. While incentives have been shown to support adoption of integrated behavioral health models on a small scale, new national efforts are only recently being evaluated for impact. At the organizational-level, funding needs to be considered for the many and, potentially evolving, implementation supports of integrated care. For example, funding may be required for, but not limited to: physical resources like office space to accommodate co-location of primary and specialist providers, technology infrastructure (e.g., changing electronic medical record systems) to support electronic communication and collaboration between providers, and staff (e.g., administrative staff, behavioral healthcare providers, patient-centered medical home specialists) who execute the new or modified practice procedures to support integrated care delivery.

#### (2) Foster meaningful collaboration with stakeholders across phases of implementation

This goal can be achieved through several strategies. One example is through establishment of a community-academic partnership [19] to plan for and consider the individual characteristics, needs and level of engagement of key individuals involved at different levels of the system integral to integrated care implementation (e.g., frontline staff, leaders and managers across different provider organisations). Another is to use a Dynamic Adaptation Process model where key stakeholders form an “implementation resource team” to help shepherd and support the implementation process [62]. A further key strategy is actively increasing levels of service user and caregiver engagement including active and strategic involvement in co-design, evaluation and implementation of integrated care programmes. There is a need to focus on genuine, meaningful ways of co-producing integrated care systems through involving service user and caregiver groups from different target population groups, particularly among populations with complex health and social care needs, to work collaboratively in partnership with professionals to improve integrated care implementation [63]. A recent systematic review [64] reported strategies for optimal patient engagement to enhance design, recruitment, a receptive context and leadership actions. Changes to care or service delivery models were more likely to derive from higher levels of patient engagement (e.g., through co-design activities) as opposed to lower levels of engagement (e.g., consultative roles). Overall, collaboration between service users, providers and researchers will likely enhance relevance, acceptability, reach and impact of integrated care programs.

#### (3) Cultivate opportunities for clear communication about integrated care implementation

Implementation climate for integrated care could be targeted by developing organizational mission statements that discuss integrated care or selecting employees for their prior experience working in integrated care settings. Another strategy is explicitly targeting leadership to support integrated care implementation [65]. Integrated care implementation leadership that could be targeted include: the amount of knowledge leaders have about integrated care components and service delivery, proactive efforts of the leader integrated care implementation or sustainment, the extent to which leaders persevere with integrated care implementation in the face of barriers, and the extent to which leaders are available, attentive, or accessible to facilitate integrated care implementation or sustainment [65]. The Leadership and Organizational Change for Implementation (LOCI) strategy is an example of a recommended implementation strategy to facilitate integrated care implementation and strengthen alignment across levels of leadership to support integrated care initiatives [66]. In addition, improving the infrastructure for collaboration through shared records and increasing opportunities for shared communication between different providers across organizational boundaries. A necessary and ongoing condition for improved communication is the clear delineation of roles and responsibilities of each individual (from patient to organizational or system leader) and process developed to support integrated care delivery.

### Strengths and limitations

There are several strengths balanced with some limitations of this research. While systematic reviews exist demonstrating the effectiveness of integrated care approaches [15], limited research exists outlining implementation strategies and/or considerations for implementation generalizable across contexts. The primary strength is our inclusion of implementation projects that vary in their phase of implementation and conduct in countries that differ in their healthcare financing and the specific user groups, systems and health outcomes targeted. This multiple, international case study design bolstered our ability to assert a proposed agenda for implementation of integrated care that could facilitate greater transfer of knowledge among research teams working in various healthcare settings. Another strength of this research is our explicit and thorough use of the EPIS framework, a widely used implementation framework, to ground the framing, analysis and interpretation of our findings. This in line with recommendations to increase the breadth and depth of implementation frameworks as an important step to advance the field of implementation science through systematic and comprehensive application of implementation science theory and frameworks [18].

Several limitations are of note. The primary limitation of this research is the snowball sampling strategy to select case studies. This sampling strategy was selected for several reasons (e.g., to align with recommendations for case study research designs, to facilitate inclusion of heterogeneous studies) but it may have resulted in the inclusion of case studies that are not representative of all integrated care implementation efforts. To mitigate this effect, we intentionally sought out integrated care researchers who were conducting implementation research in a range of high income and low-to-middle income countries that were focused on a variety of service systems and patient populations. In addition, some potential contributors were excluded from inclusion if it was felt that they would lead to excessive redundancy between case studies. We believe that we have provided a fairly broad range of examples. Though the sampling effort may have limitations, we believe the conclusions presented have broader applicability beyond the integrated care implementation efforts included in this paper. Within this effort to include a range of integrated care examples, we acknowledge that we sought out researchers who are leading or who are involved in integrated care program implementation. Commonly, integrated care programs do not involve researchers and are often implemented in care contexts that limit the feasibility of engaging researchers and community members in community-academic partnerships. An additional limitation is that we did not include an independent evaluator to assign or review the work described in each case study to the specific domains of the EPIS framework. However, we employed an iterative qualitative analytic approach that provided all research leads of the case studies and the lead EPIS framework developer the opportunity to review and refine the operationalization and application of the EPIS domains and phases to each case study.

## Conclusion

This multiple case study design highlights research from five countries in pursuit of implementing integrated care models in a range of service systems for various patient groups. Synthesis of case study descriptions revealed common themes related to the outer, inner and bridging/innovation contexts that informed our proposed agenda to advance the research in integrated care implementation. The primary goals within our proposed agenda relate to 1) the role of funding at multiple levels of implementation; 2) fostering meaningful collaboration with stakeholders across phases of implementation; and 3) clear communication about integrated care implementation.

## Supplementary information

**Additional file 1.** Full Text Case Studies. These are the full case studies based on the standard template (see Table 1) that were submitted to the core research team (NS, ES, MS).

## Data Availability

Not Applicable.

## Abbreviations

ASD: Autism spectrum disorder
ATTAIN: Access To Tailored Autism INtegrated Care
EPIS: Exploration, Preparation, Implementation, Sustainment (EPIS) Framework
HMO: Health Maintenance Organization
SPECTRA: Scaling up Care for Perinatal Depression for Improved Maternal and Child Health
PCMH: Patient Centered Medical Home Model
FCC: Family Care Center
