# Supplementary material for: Comparative case studies in integrated care implementation from across the globe: a quest for action
Source: BMC Health Serv Res. 2019 Nov 27;19:899. doi: 10.1186/s12913-019-4661-5 (PMC6882190; doi:10.1186/s12913-019-4661-5)
Supplement: Supplementary file 1 — Additional file 1. Full Text Case Studies. These are the full case studies based on the standard template (see Table 1) that were submitted to the core research team (NS, ES, MS). [file 12913_2019_4661_MOESM1_ESM.docx]

1. **Integrated Care for Older Adults with Frailty in South London, UK (Exploration-Preparation)**

To meet the healthcare needs of the growing number of older people, this project targets implementation of integrated care for community dwelling older adults with frailty [26] in South London, UK to co-design a model of integrated care to improve patient, provider, and service outcomes. The implementation context centers on two London boroughs that are densely populated (over 300,000 local population), have high levels of deprivation and are ethnically diverse.

Outer and inner context factors within the Exploration and Preparation phases of EPIS have guided efforts to examine key context factors influencing the design and early adoption of integrated care for older adults with frailty. For example, key outer context factors included: the role of national and local policy drives and funding to improve integrated care delivery for cohorts of patients at high risk of hospital admission [28] and early stakeholder engagement and relationship building with local care providers, service users and caregivers. Key inner context factors include: organizational characteristics (i.e. role specialization, knowledge skills, expertise, values) and leadership qualities required to enable early adoption and identification of leaders in the system to champion adoption and delivery of integrated care.

We identified a number of challenges, successes and lessons learned. First, providers working in different parts of the health and social care system largely shared a common understanding that integrated care for older adults with frailty involves different providers working in effective multi-disciplinary teams across different care organizations and sectors (i.e., EPIS bridging factors) to deliver patient-centered, holistic, and coordinated health and social care. A number of health and social care professionals perceived that there were improved relationships between providers working in health and social care, but in practice there was still limited coordination of care and joint working of providers across health, social and voluntary care sectors as a strategy for integrated care for this population. Service users and carers found it difficult to conceptualize integrated care, but some similarly understood it as improved coordination of health and social care services, whilst others viewed integrated care as continuity of care with a trusted professional who knew them and having the right information and resources (e.g. social capital from family members and carers) to access and navigate the system. For example, one woman in her late sixties with frailty and multimorbidity commented on the importance of relational continuity of care with her General Practitioner (GP):

*“He (GP) knows me, he knows what’s wrong with me, he knows that if I’m saying, ‘This pain has changed’, then he knows there’s a reason it’s changed and he will look into it or he’ll send me to the hospital. You know, he knows me. I’m not a stranger, he’s not a stranger to me.”*

Perceived barriers to implementation of integrated care among stakeholders mainly reflected contextual factors related to organizational (e.g. siloed working, lack of pooled budgets, limited co-location of teams, top-down approaches dominate), system or structural factors (e.g. reduced access, coordination and continuity of care, limited access to shared patient records, limited funding); and individuals (e.g. patient complexity, variations in attitudes of managers, leaders and frontline staff, lack of champions). For example, one physiotherapist said:

*“[T]he real barrier to good integrated care is the healthcare and, well, professionals in general who are looking after patients don’t really trust each other. I think one of the lynchpins to good integrated care is actually having a single assessment process so that the patient isn’t being asked hundreds of questions all the time and that people aren’t coming in and doing the same thing over and over again. That’s what the patients will tell you, they get really sick and tired of having to repeat the same information over and over again.”*

These outer and inner context factors will inform selection of several implementation strategies to improve the delivery and implementation of models of integrated care for older adults with frailty and their carers. These include: identifying and mapping out multi-domain barriers and facilitators to implementation based on our multi-stakeholder qualitative study to evidence based implementation strategies; synthesizing and integrating findings from the systematic review and evidence synthesis studies, applying for funding to co-design and evaluate the effectiveness and implementation of a model of integrated care for this population, and engaging service users and caregivers as active participants through local consensus discussions.

1. **Access to Integrated Care Tailored for Children with Autism Spectrum Disorder in California, USA (Exploration-Implementation)**

The goal of the “Access To Tailored Autism INtegrated Care” (ATTAIN) study [29] is to adapt and implement a behavioral health integrated care model between pediatric primary care and mental health care, for children with autism spectrum disorder (ASD). This target group was selected because they experience high rates of co-occurring mental health conditions that result in complex medical presentations and challenging health care management [67].

The ATTAIN Study is currently in the Exploration phase and will span through Preparation to the Implementation phase. The key inner context factors are being measured through a mixed-methods needs assessment involving organizational health care leaders, pediatric primary care providers, and caregivers of children with ASD. At the organizational level, the primary targets are readiness and capacity for change and the flexibility and utility of electronic health record systems in the various health care organizations. At the provider and caregiver level, key inner context targets include knowledge of co-occurring mental health problems in children with ASD and perceptions about integrated care, and interest in modifying certain integrated care procedures (screening and referral) for children with ASD. Across levels, the key inner context factor targeted is the “fit” between ATTAIN and the organizations in which it is implemented, the providers who use ATTAIN, and the caregivers and children with ASD who receive ATTAIN. Informed by these inner context factors, the primary implementation strategy applied to ATTAIN implementation is establishing a community-academic partnership to promote successful implementation and pediatric primary care provider training in tailored mental health screening and referral practices established by the ATTAIN model of integrated care. Ultimately, the implementation outcomes that will be targeted and evaluated in a later staged open trial feasibility pilot test are ATTAIN feasibility, acceptability, uptake [68] and efficiency of implementation.

Based on preliminary qualitative data, several successes, challenges, and lessons learned have been illuminated. A key driver of ATTAIN implementation is the degree to which integrated care already exists and the ability to adapt components of healthcare delivery within the limitations of the organizational structure of the healthcare organization. This is consistent with the EPIS notion that adaptation may be needed at outer or inner context, or to the integrated care model itself. One pediatric provider highlighted how a structural factor (presence of a shared electronic health record system) can facilitate coordination of care,

*“one advantage that I see is when we make referrals…within the network. Our mental health or behavioral health providers have access to the medical records so they can take a look and see …what our thinking is, what observations we made, what actions we’ve taken. And that’s nice.”*

However, the current healthcare landscape in primary care is not setup to support or incentivize pediatric primary care providers to attend to mental health concerns. As one organizational health care leader said,

*“I believe you would find that there would be a huge sense of willingness to own this issue if we got paid for it. A lot of what we do is uncompensated care.”*

Furthermore, multiple organizational leaders and primary care providers voiced support for enhanced care coordination as a promising but uncertain solution,

“*In an ideal world I think what would really help is an interdisciplinary team. You know, where you do have that Care Coordinator, you have the development, the mental health, and the provider, like, working very closely together.”*

These needs assessment results highlight the dynamic interplay between outer and inner context factors that need to be proactively considered at the early stages of implementation.

1. **Perinatal Depression Screening and Treatment in Can Tho, Vietnam (Exploration-Implementation)**

This project aims to implement an integrated care approach (collaborative care) to improve screening and care for common perinatal mental health disorders in Vietnam, a lower income country with a significant focus on the delivery of healthcare through public health services. The Can Tho University of Medicine and Pharmacy is the only public health service provider for this region (Can Tho in South Vietnam) and provides support to the regional public health delivery system. The public health system focuses on the commune health clinic (CHC), which is where the majority of public health care is provided. Linked to each CHC is a District health center and a regional hospital which provide higher levels of support through coordination and specialty services. Each CHC also provides support to village or hamlet community health care workers (collaborators).

This project is currently in the Exploration and Preparation phases of the EPIS framework. The overarching implementation strategy used for this project was a community-academic partnership through a participatory developmental approach. The key outer context factor that was an implementation driver was the National Mental Health Initiative for primary care, which identified depression as a target. The key inner context factors targeted were organizational characteristics of the CHCs (e.g., infrastructure to support collaborative care, implementation climate, volume of prenatal and pediatric patients) and individual characteristics (e.g., training and professional background of CHC providers, knowledge of perinatal depression).

These inner context factors were explored through descriptive and qualitative data collected through review of health care delivery records and interviews or focus groups with providers at care delivery sites. Descriptive and qualitative data were collected through review of health care delivery records and interviews with providers at care delivery sites and in focus group setting. Among 22 CHCs that were potential targets of this implementation effort, staffing included one midwife, one to two primary care physicians, a traditional medical provider, a physician assistant, and a record administrator. One physician at each site was assigned to lead the efforts to carry out the national mental health care program. Each site had access to a medical supply/pharmacy. Sites ranged from 150-200 prenatal patients annually with approximately 4000 prenatal patients annually. In the prior year there were no women identified with perinatal depression at any of the clinical sites or referred to mental health providers. Interviews were carried out by first exploring current efforts to identify and treat perinatal depression followed by a review of symptoms, epidemiology, and outcomes related to untreated perinatal depression. The evidence based collaborative care model of perinatal depression service delivery was described to stimulate discussion. Interviews with staff at individual commune health centers and in focus group setting indicated that there was a lack of knowledge of the symptoms of perinatal depression. Once reviewed participants indicated recognition of this condition as common. There was a high level of perceived need and alignment with the goals of the health settings but a lack of training and procedures to allow for screening and care of perinatal depression.

Based on the results of this exploratory process, a pilot implementation of collaborative care for perinatal depression in one CHC was planned. Needed linkages were provided to a supporting District Health center and Hospital as well as to community health workers in hamlets and villages. Elements of collaborative care identified for the pilot study included systematic screening using a version of the Patient Health Questionnaire (PHQ-9), validated for use in Vietnamese maternal care, the use of care management, use of a patient registry, weekly specialty psychiatric case review of patients on the registry using telephonic communication, and the training of community health workers to deliver supportive counseling in collaboration with the health providers at the health centers. An existing federally supported effort to initiate screening for depression in CHCs was identified as an opportunity to leverage regional resources for the pilot. Obstacles include the need to find further funding for the participation of external experts and health services researchers to support the pilot and evaluation with subsequent decisions regarding elements of the collaborative care model that could be implemented.

Through this work, we have observed important implementation themes that have impacted our integrated care effort. A critical implementation driver was the existing federally supported effort to initiate screening for depression in CHCs. This national policy initiative was an opportunity to leverage regional resources for the pilot implementation study. The primary challenge to implementation is identification of additional funding sources for the participation of external experts and health services researchers to support the pilot and evaluation.

1. **Integrated Care Following Mental Health Insurance Reform in Israel (Implementation phase)**

Beginning soon after the establishment of the State in 1948, Israel developed a comprehensive mental health care system which provided care through psychiatric hospitals and government-financed mental health clinics spread throughout the country [69]. This system existed in its own administrative structure, primarily through the Israeli Ministry of Health, apart from the four major independent health maintenance organizations (HMOs or sick funds) which provided universal healthcare services to all the citizens and residents of the country. Limited behavioral health integration occurred with mental health services in the HMOs, particularly social work and to some extent, psychiatry, which were available in a portion of the primary care clinics and general hospitals.

The provision of general health services and mental health care through separate organizational structures changed in 2015 when the responsibility for the provision of mental health care was transferred from the Israeli Ministry of Health to the four major HMO health plans. These plans are now mandated to provide mental health care and do so through a mix of “salaried professionals, contracted independent professionals, and services purchased from organizations (including the ministry’s mental health clinics) [Commonwealth]...” The basket of services, or benefit package, is wide-ranging and includes outpatient and inpatient care, psychotherapy, medications, other treatments. Integration with primary care is expanding and is expected to further improve as the HMOs role out their implementations. Mental health services are now intended to be supplied to the entire population of Israel (citizens and residents). This mental health reform intends to increase the quality, availability, and accessibility of mental health services, reduce stigma toward those coping with mental illness, and consolidate funding [33].

One clinic that is actively implementing, and sustaining, integrated behavioral health services in Israel is the Tivon General Sick Fund (*Clalit*) clinic, located in the Town of Kiryat Tivon in the Haifa district of northern Israel. Kiryat Tivon is a town of approximately 18,000 residents who are primarily working-class and middle-class Israeli Jews, The Tivon Clalit clinic is the largest of the primary care health facilities in the town and provides services to several thousand patients.

Numerous outer and inner context factors influenced implementation of integrated behavioral health in the Tivon clinic. Major outer context factors that facilitated implementation are government and HMO policies and funding since the acceptance of the 2015 mandate. Financing came directly from the Ministry of Finance to the HMOs, including Clalit Health Services (not directly to the Tivon clinic). Cultural stigmas around mental health services also influenced implementation. For example, religious perceptions of the “science-magic” divide. Stakeholder commitment and engagement, together with organizational inertia, were key inner context factors that facilitated implementation. In preparation for implementation, the HMOs hired trained and professional personnel including medical staff, psychiatrists, therapists and social workers. Many of these individuals worked for the Ministry of Health and moved to the HMOs. In addition, the Tivon clinic designated a room to be used by the behavioral health providers to facilitate integration.

Throughout implementation of integrated behavioral health care in the Tivon clinic, several challenges emerged. While there was hope for increased communication and cooperation between the primary care providers and the behavioral health staff, particularly due to sharing clinic space, this collaboration was never realized. There are two potential reasons for this unrealized hope: (1) the Tivon clinic was only provided with one psychiatrist despite promises of also being provided with a social worker and therapist, and (2) psychiatrist services at the Tivon clinic were only offered once every two weeks, precluding the psychiatrist from joining weekly staff meetings with other clinic providers. The limited behavioral health services offered at Tivon resulted in several adverse outcomes including poor linkages between the primary care providers and the behavioral health team, insufficient availability of, and poorer quality, behavioral health services, long wait times to be seen by the psychiatrist, and inability to provide services for specific populations (e.g., patients with eating disorders).

While the Tivon clinic serves as one example of the implementation of integrated behavioral health care in Israel following reform, broader challenges and successes have been identified. Additional challenges remain regarding availability of services—which is largely dependent on geographical factors, such as the concentration of mental health professionals in the large cities, as well and some inequities in distribution based on social economic and socio-political factors. There also remain issues of coordination and connection between services, particularly regarding the care of children.  Despite ongoing challenges, successes should be acknowledged. These have included, for example, the transfer of responsibility for some of the less serious mental health illnesses from psychiatry to primary care. Additionally, relative integration of physical and mental health services has been achieved in some clinics across Israel [36-38], with the elimination of prior institutional barriers.

**5. Scaling up Care for Perinatal Depression in Primary Care, Oyo State, Nigeria (Implementation phase)**

The Scaling up Care for Perinatal Depression for Improved Maternal and Child Health (SPECTRA) project used a task sharing approach to integrate care for perinatal depression into primary maternal care where the largest proportion of Nigerian women receive maternal and child health services. The study context is Oyo State in the Southwest geopolitical zone of Nigeria. Each local government area (of 33) has about 10-14 primary health care centres. Health services are mainly provided by non-physician primary health care providers consisting of nurses, community health officers and community health extension workers. This project was carried out in 11 local government areas (5 urban and 6 rural) located in and around the state capital, Ibadan. Even though the national mental health policy makes provision for mental health services to be delivered in primary health care, in reality, mental health services are delivered in large tertiary institutions (e.g., university teaching hospitals).

The SPECTRA project is currently in the implementation phase of the EPIS framework [10]. The key outer context driver of implementation was heightened state policy attention to maternal mental health care. This led to the development of a sustainable model for training primary care workers to implement the existing National Mental Health policy on maternal mental health care. Inner context factors impacting the implementation of integration include an initial resistance to change by frontline primary health care workers, limited knowledge of perinatal depression screening, high workload, and the absence of consistent leadership in the form of supportive supervision for the frontline providers to facilitate the delivery of evidence-based care for perinatal depression.

Informed by the results of the formative studies, training and implementation manuals were subsequently developed. This included the adaptation of the World Health Organization Mental Health Gap Action Programme Intervention Guide (mhGAP), to reflect the structure and profile of the country’s health system, including the configuration of its personnel. The mhGAP is a tool developed specifically to assist non-specialists in the diagnosis and evidence-based treatment of some priority mental disorders, including depression. Considering that one of the key problems in this resource-constrained setting is the scarcity of mental health professionals, our primary implementation strategy was the use of a cascade training approach, where psychiatrists (master trainers), trained senior level primary health care workers (including primary care physicians, senior nurse/midwives and senior community health workers) as non-specialist trainers who then provided training to the frontline primary health care workers. These non-specialist trainers successfully trained 198 frontline providers in a series of 2-day training workshops (didactic lectures and role plays), with each workshop being conducted by a pair of trainers for 20-25 trainees and with a master trainer sitting in to observe and offer support when required. The training was focused on the use of the mhGAP-IG depression module for the identification and treatment of the condition among perinatal women. Following the training, and to assess the impact of routine screening on the detection of perinatal depression, we introduced a brief screening instrument (the 2-item patient health questionnaire [PHQ-2]) to be used by the providers during their routine assessment of perinatal women.

Even though the cascade training was successful, resulting in measurable improvement in the knowledge about and attitude to depression by the trainees, and the attendant increase in skilled mental health workforce capacity, a persisting challenge was the low detection rates of perinatal depression. The detection rate for perinatal depression rose following training and further with the introduction of the use of PHQ-2 but remained low at about 14%. In response to the persisting low detection rates and to improve care delivered, we incorporated a structured supportive supervision program at the clinics. The trainers visit each clinic twice a month to observe, debrief and provide additional training and support for difficult cases. While the providers are able to deliver the interventions with good fidelity and there was evidence that, once detected, delivery of depression care had improved, the challenge remains how to improve identification for depression.

1. **Midwifery Continuity of Care Model for Women at increased risk of Preterm Birth in South London, UK (Implementation & Sustainment phases)**

A recent Cochrane review found that women who receive continuity of care by a named midwife or a small group of midwives throughout pregnancy, birth and the postnatal period are 24% less likely to experience a preterm birth, more likely to have better maternal and infant outcomes, report positive experiences of care and use healthcare resources more effectively [70]. A hybrid type-2 effectiveness-implementation pilot trial [42] was designed to test the impact of a new integrated care pathway that combines midwife continuity of care (in both community and hospital settings) with rapid referral to a specialist obstetric preterm birth clinic during pregnancy and throughout the postpartum period for women who are at increased risk of preterm birth in South London, UK. Approximately 30% of the population in this borough are from Black and Minority Ethnic groups and levels of deprivation are higher than the national average [71]. Deprivation is associated with increased rates of stillbirth, preterm birth, low birth weight babies, neonatal deaths and infant mortality [72]. Therefore, women in this area are at greater risk of these outcomes than women from more affluent areas. The aim of this ongoing study is to determine whether this new model of integrated care is feasible, improves pregnancy experience, as well as improves clinical and process outcomes for these mothers, babies and families. To date 328 women have been recruited to the trial and randomized to either standard care or continuity of midwifery care.

The implementation and sustainment phases of the EPIS framework guided identification of contextual factors facilitating or hindering implementation efforts. During the exploration and preparation phases between 2014 and 2016 researchers, clinicians and managers, public health consultants, commissioners and service users in South London discussed a potential collaborative research opportunity to support the reduction of preterm birth, as a leading cause of death in children in their area. Considering the evidence, it was hypothesised that providing a midwifery continuity of care model to women with risk factors for preterm birth would have an impact on the number of preterm births and quality of care and experience for local women. Key inner contextual factors identified to facilitate short- and longer-term implementation included seeking organizational commitment and developing a shared vision at local and national levels; identifying and enhancing leadership and visibility, whereas factors hindering implementation included a lack of tangible financial incentives and significant workforce staffing shortages. In terms of key outer contextual factors facilitating implementation, this included the national maternal policy “Better Births” [43] to increase continuity of care; enhanced tariffs from local clinical commissioner groups and developing a robust network between midwifery services and other external organizations. In contrast, key external contextual factors hindering implementation included a lack of research culture in this maternity service, and mimetic pressure from competing organizations who have already implemented similar models of care.

The identified inner and outer contextual factors expected to influence the implementation process informed the selection of more than 20 evidence-based implementation strategies drawn from the Expert Recommendations for Implementing Change (ERIC) project [44]. These included conducting a local needs assessment that highlighted the need for midwifery continuity of care to reduce perinatal mortality and morbidity and building a coalition to cultivate relationships and co-develop health programmes across partners. The implementation of a midwifery continuity of care model involved a complex, large-scale transformation of the organisation of maternity care services. Some of the challenges to implementation and scale up may be related to inner context factors such as staff recruitment, change of working patterns, midwife concerns about burnout, manager concerns about cost, organizational disruption and how to manage change, and a lack of clarity around which particular populations to target when whole system change is not possible.

Key achievements have been the early and ongoing engagement with the commissioners of maternity services in project design who have provided additional financial support for a clinical lead and commissioning the planned service in the contract with the hospital. Local commissioners highlighted the value of stakeholder engagement of the integrated care research process stating,

*“We are very proud to be part of this exciting collaboration, which is an example of true partnership working… We have high hopes for the learning that will be generated from the trial in terms of improving support for women at risk of pre-term birth, as well as for other women accessing the maternity services…”*

Other successes include a national maternity policy plan which aims to increase continuity models of care nationally, and the enthusiasm of all involved in the project has attracted midwives from outside the organisation. In addition, the project has received an NIHR ‘*Most Innovative Collaboration*’ Award.

1. **Patient-Centered Medical Home Model for Adults and Children to Promote Health and Wellbeing in Rhode Island, United States (Sustainment phase)**

The Patient-Centered Medical Home Model (PCMH) approach is an innovative model for integrated primary care services [73], and efforts across the USA and Canada [73, 74] are targeting the transformation of primary care clinics into PCMHs. The PCMH emphasizes ongoing patient-physician relationships, improved care access, and provision of comprehensive care by addressing all health care needs at all stages of life [51, 74, 75]. The provision of integrated care through implementation and sustainment of PCMH model of service delivery will be discussed using the Family Care Center (FCC) at Memorial Hospital of Rhode Island, USA, as an example. The FCC is a primary care clinic operating as a PCMH. The FCC offers integrated multidisciplinary services that include gynecology/colposcopy, behavioral health, osteopathic manipulative medicine, family planning, medication assisted treatment for addiction, geriatrics, nutrition, high-risk obstetrics, and pediatric development.

The FCC’s transformation into a PCMH is now in the Sustainment phase of the EPIS framework [10]. Several outer and inner context factors were key in facilitating the FCC’s progression into the Sustainment phase of PCMH implementation. Key outer context factors include a history of transdisciplinary, and legislative, initiatives to improve United States health care and patient health outcomes. A notable example includes the development and endorsement of the Joint Principles of the PCMH in 2007 by the American Academy of Family Physicians, the American Academy of Pediatrics, the American College of Physicians, and the American Osteopathic Association, PCMH [51]. Another notable example includes the Patient Protection and Affordable Care Act of 2010—comprehensive health care reform law—that provided further support for the implementation of the PCMH through several provisions directed at the establishment and promotion of the PCMH. Several inner context factors were also important in facilitating the FCC’s progression into the Sustainment phase. The Department of Family Medicine at Brown Medical School housed faculty members with leadership roles in national organizations in academic family medicine that facilitated the collaboration and knowledge sharing between the FCC and Patient-Centered Primary Care Collaborative regarding the future of family medicine. These faculty members then obtained grant funding to support the emerging PCMH movement in Rhode Island and enrolled the FCC in a statewide chronic care collaborative in 2003. Another important inner context factor in facilitating the FCC’s progression into the Sustainment phase of PCMH implementation is that Brown Medical School curriculum has been adapted to include additional didactic and experiential training on the PCMH.

The connections between the FCC, the Department of Family Medicine at Brown Medical School, and national organizations like the PCPCC, together with curriculum changes, iterative research, fidelity monitoring, and adaptations based upon data were key implementation strategies that facilitated the transformation and sustainment of the PCMH model of service delivery at the FCC. An additional key contributor to the transformation and sustainment of the PCMH model of service delivery at the FCC was the innovation-values fit [76]. FCC faculty and resident physicians believed the PCMH model of service delivery was harmonious with the values of family medicine. For example, one FCC faculty physician stated:

“*I sort of feel like there’s what we emotionally think a patient-centered medical home is, which a lot of family docs say, ‘Well, we were already doing that. We were already patient-centered, we already had good access, and we take good care of our patients, and we’re very coordination of care oriented.’ I think I’ve come to think of [the PCMH] as an attempt to highlight the best practices in primary care.*”

While the FCC has been able to sustain the PCMH model of service delivery, areas for improvement reported by FCC faculty and resident physicians include ongoing challenges of limited time, feeling/being under resourced, limited staffing, and payment systems that do not adequately support the PCMH model. For example, another FCC faculty physician summarized these challenges stating:

“*… everybody’s too busy. There isn’t enough staff. The jobs are defined in a way that doesn’t have that kind of flexibility. It’s from the moment you check in to the moment you leave it’s full of tasks, and there’s no room for coming to a meeting to do this, even if you—even if you wanted to…Can they really carry the weight of the practice on their shoulders in terms of fixing systems.*”

Despite these challenges, several successes deserve acknowledgement. Many faculty and resident physicians described the widespread knowledge of the PCMH within the FCC, and the integration of some structures and processes to facilitate integration, as successes.
